# Wearable‐Derived Diurnal Alignment Between Physical Activity and Device Temperature Predicts Future Disease and Mortality Risk

**DOI:** 10.1002/advs.76217

**Published:** 2026-06-19

**Authors:** Han Chen, Jiahe Wei, Jonathan Cedernaes, Christian Benedict, Athanasios Tsanas, Zhi Cao, Xiao Tan

**Affiliations:** ^1^ Department of Psychiatry, Sir Run Run Shaw Hospital Zhejiang Key Laboratory of Clinical and Basic Research for Psychiatric Diseases and School of Public Health Zhejiang University School of Medicine Hangzhou China; ^2^ Department of Medical Sciences Uppsala University Uppsala Sweden; ^3^ Department of Medical Cell Biology Uppsala University Uppsala Sweden; ^4^ Department of Pharmaceutical Biosciences Uppsala University Uppsala Sweden; ^5^ Usher Institute University of Edinburgh Edinburgh Scotland UK; ^6^ Department of Clinical Neuroscience Karolinska Institutet Stockholm Sweden

**Keywords:** circadian disruption, diurnal alignment, phenome‐wide association study, prospective cohort, wearable accelerometry

## Abstract

Circadian rhythms coordinate physiology with the 24 h light‐dark cycle, and their disruption contributes to diseases spanning metabolic, cardiovascular, and neuropsychiatric domains. Whether the temporal coherence between wearable‐derived activity and temperature rhythms predicts long‐term health outcomes in free‐living humans remains unknown. Here, analyzing week‐long concurrent wrist‐worn acceleration and device temperature recordings from approximately 90,000 UK Biobank participants (median age 63 years), we decompose the circular cross‐correlation between behavioral and device temperature signals into three alignment features, including 24 h coupling strength (M_24_), phase deviation from expected antiphase (D_24_), and 12 h harmonic magnitude (M_12_). Over 7–11 years of prospective follow‐up, higher M_24_ is associated with lower risk of type 2 diabetes, cardiovascular disease, depression, sleep apnea, and all‐cause mortality, whereas higher D_24_ is associated with increased cardiometabolic risk. Higher M_12_ was associated with a lower risk of gastrointestinal and psychiatric conditions. Technical replication in the SHARE cohort supported the portability of the feature‐extraction framework across device protocols. These findings highlight wearable‐derived cross‐domain diurnal alignment as a scalable, prospective predictor of disease risk, with potential implications for population health surveillance.

## Graphical abstract

Wearable‐derived diurnal alignment between physical activity and device temperature, decomposed into 24 h coupling strength (M_24_), phase deviation (D_24_), and 12 h harmonic magnitude (M_12_), is examined in approximately 90,000 UK Biobank participants. Stronger alignment prospectively predicts lower risk of cardiometabolic, neuropsychiatric, and gastrointestinal disease and mortality, establishing a scalable digital biomarker for population health surveillance.

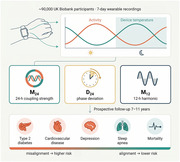

## 1 Introduction

The mammalian circadian system coordinates physiological rhythms through a hierarchical network of molecular oscillators [1]. Recent reviews emphasize that circadian timing is not merely a background physiological process but an organizing principle linking genetic regulation, environmental cues, disease risk, and therapeutic response [2, 3]. The master pacemaker, located in the hypothalamic suprachiasmatic nucleus (SCN), transduces photic information to synchronize peripheral oscillators distributed across metabolic tissues [3]. While the SCN entrains primarily to the external light‐dark cycle, extra‐SCN and peripheral clocks integrate diverse zeitgebers, some of which are SCN‐driven. These include the 24‐h timing of other input, from feeding patterns, physical activity, exogenous substances (e.g., medications), body temperature fluctuations, to social interactions [4]. This multi‐oscillator architecture necessitates precise temporal coordination among tissue‐specific clocks to maintain physiological homeostasis.

Core body temperature (CBT) represents a robust circadian output regulated by central thermoregulatory circuits [5]. Under entrained conditions, CBT exhibits a 24 h oscillation with the approximate amplitude of 1.0°C, reaching its nadir during the biological night (around 2–3 h before habitual wake‐up) and acrophase in the early biological evening [6]. Distal skin temperature demonstrates an antiphasic oscillation, increasing nocturnally to promote core‐to‐shell heat dissipation [7]. Exercise‐induced thermogenesis during the active phase potentiates the CBT amplitude, thereby enhancing the thermal zeitgeber signal that entrains peripheral oscillators [8, 9]. Contemporary environmental challenges, including shift work, social jet lag, and temporal feeding disruption, generate circadian misalignment by decoupling central and peripheral oscillators [3, 10, 11]. This internal desynchrony between tissue‐specific clocks represents an emerging mechanism linking circadian disruption to metabolic dysfunction and disease pathogenesis.

Wearable devices offer large‐scale opportunities to observe 24 h rhythms in physiology in free‐living individuals [12, 13, 14]. Wrist devices can record high‐resolution fluctuations in activity and concurrent device temperature, which provides an imperfect proxy for local wrist skin temperature [15]. Previous wearable studies have typically examined single modalities like acceleration or device temperature in isolation, rather than examining inter‐rhythm phase relationships, and employ conventional cosinor analysis that inadequately captures the complex waveforms characteristic of human circadian outputs [16, 17].

**FIGURE 1:**
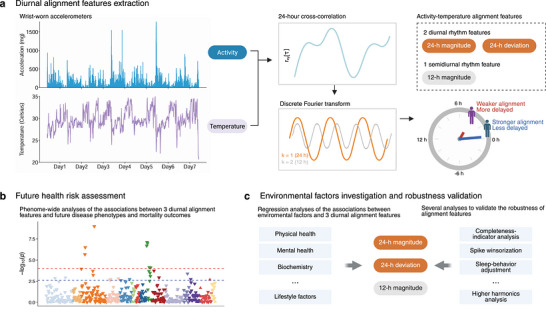
Overview of the diurnal alignment analysis framework. (a) Extraction of activity‐temperature alignment features from week‐long wearable recordings. Representative 7‐day recordings of physical activity and device temperature from approximately 90 000 UK Biobank participants. The 24 h circular cross‐correlation function *r_xy_* [τ] captures the antiphase relationship between activity and device temperature rhythms. Discrete Fourier transform of the cross‐correlation function reveals dominant periodicities at 24 h (*k* = 1) and 12 h (*k* = 2). Three alignment features are extracted: 24 h magnitude (M_24_) representing diurnal coupling strength, 24 h deviation (D_24_) representing phase angle from expected antiphase, and 12 h magnitude (M_12_) capturing semidiurnal rhythmicity. The circular plot (right) illustrates these features, with vectors showing diurnal alignment strength and phase relationships. (b) Phenome‐wide association analysis reveals disease‐specific patterns across alignment features. (c) Investigation of upstream factors influencing diurnal alignment, and robustness validation of alignment features. Regression analyses identified associations between upstream environmental factors and diurnal alignment features (left). Analyses validated the robustness of alignment features (right).

This study applies a joint acceleration‐device temperature alignment framework to approximately 90,000 upper‐middle‐aged UK Biobank participants with week‐long wearable recordings (Figure 1). We pursue three main objectives. First, we construct alignment features from concurrent acceleration and device‐temperature data and evaluate their reliability and validity. Second, we conducted a comprehensive phenome‐wide association study (PheWAS) exploring statistical relationships with a wide range of health outcomes and tested whether baseline alignment predicts future mortality from all causes and cardiovascular disease (CVD) specifically. Third, we identify upstream environmental factors associated with alignment features.

## 2 Results

### 2.1 Description of the Study Participants

We analyzed 89,309 UK Biobank participants who met quality control (QC) criteria from an initial sample of 103,610 who had provided accelerometry data (for QC and flow chart, see Figure S1). Participants had a median age of 63 years (interquartile range (IQR): 56–69) at accelerometry assessment, with 56.5% being female and 97.1% being White (Table S1).

### 2.2 Derivation and Validation of 24‐h Alignment Features

Participants provided continuous wrist‐worn acceleration and device temperature measurements for a median of 7 days. From these data, we derived three key metrics that capture different aspects of how daily activity patterns align with device temperature rhythms. Specifically, we compute circular cross‐correlations between standardized daily patterns of acceleration and device temperature, then decompose the correlations using Fourier transforms to isolate 24 and 12 h harmonic components, which we together refer to as components of 24‐h‐alignment (hereafter denoted 24hAlign) (Methods). The first metric measures the strength of 24 h coupling between activity and device temperature, denoting 24‐h magnitude (M_24_). A high M_24_ value near 1 indicates strong regular coupling where activity and device temperature maintain a stable relationship, approaching 0, suggesting weak or irregular coupling. The second metric quantifies how far the temperature rhythm deviates from its expected timing relative to activity, denoting 24‐h deviation (D_24_). In healthy physiology, device temperature should peak approximately 12 h offset from activity peaks, reflecting nighttime warming for heat dissipation versus daytime activity‐related heat dissipation. The D_24_ measures the absolute phase deviation from this 12 h offset in hours. The third metric, 12‐h magnitude (M_12_), captures the strength of twice‐daily or semidiurnal patterns that may reflect biphasic activity patterns common in human behavior. Before decomposing into these frequency‐specific metrics, we examined the raw cross‐correlation function (*r_xy_* [τ]) between activity and device temperature. Across the population, the median lag τ at which device temperature most strongly correlated with activity was 11.4 h (Figure S2a), close to the theoretical 12 h antiphase relationship we hypothesized. The distribution of 24hAlign features across the population revealed substantial inter‐individual variation (Figure S2b–d). The M_24_ showed a median of 0.3 with a right‐skewed distribution. The D_24_ exhibited a median of 0.6 h with a long tail extending to several hours of misalignment. The M_12_ displayed a strongly right‐skewed distribution with a median of 0.1, indicating that pronounced semidiurnal patterns are present but not universal across individuals. Bootstrap validation confirmed these metrics were numerically stable within individuals (Figure S3). Recomputing features at different time resolutions from 30 s to 5 min yielded nearly identical results, and alternative standardization methods produced consistent estimates (Figure S4). Considering activity and temperature are expected to be highly seasonally variable, we computed season‐adjusted 24hAlign features across all participants and produced consistent results (Figure S5). Cross‐spectral decomposition (Note S1) showed the 24‐h harmonic (*k* = 1) captured a median 93.7% of cross‐correlation energy and was statistically significant in 85.5% of participants, with the 12‐h harmonic (*k* = 2) adding 3.7% and significant in 54.2% of participants (permutation test with Benjamini‐Hochberg false discovery rate (FDR) *q* < 0.05; Figure S6). We focused on *k* = 1 and *k* = 2 for parsimony and interpretability, whilst higher harmonics may be biologically meaningful in certain subgroups. Associations with the 8‐h (*k* = 3) and 6‐h (*k* = 4) harmonics are reported as exploratory analyses below. These findings establish the technical reliability of our 24hAlign measurement approach.

**FIGURE 2:**
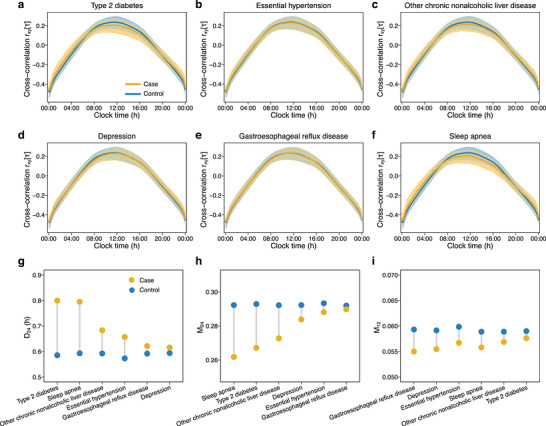
Activity‐temperature cross‐correlation patterns trajectory. (a–f) 24 h cross‐correlation functions for incident disease cases (orange) and matched controls (blue) across six representative conditions: type 2 diabetes (a), essential hypertension (b), other chronic nonalcoholic liver disease (NAFLD) (c), depression (d), gastroesophageal reflux disease (e), and sleep apnea (f). Lines represent group median with shaded areas indicating interquartile range (25th to 75th percentiles). (g–i) Quantitative comparison of the three 24hAlign features (M_24_, D_24_, M_12_) between cases and controls for the same six diseases. Each panel shows one feature across all six disease conditions. Points represent group median.

We compared the cross‐correlation patterns between participants with and without specific conditions (Figure 2). We displayed six illustrative exemplars chosen a priori to cover circadian‐relevant domains (metabolic, circulatory, digestive, psychiatric, and neurologic). Within each domain, we selected the FDR‐significant phenotype with the largest number of incident cases and the largest standardized effect on 24hAlign features. Across six representative diseases, including type 2 diabetes, essential hypertension, other chronic nonalcoholic liver disease (NAFLD), gastroesophageal reflux disease (GERD), depression, and sleep apnea, we observed consistent disruptions in alignment patterns. Disease cases showed flattened cross‐correlation trajectories compared with matched controls, indicating weaker coupling between activity and device temperature rhythms (Figure 2a–f). Type 2 diabetes and sleep apnea showed the largest phase deviations, with D_24_ substantially higher than controls (Figure 2g). Sleep apnea demonstrated the most profound reduction in coupling strength with markedly lower M_24_ values (Figure 2h). GERD and depression showed the most profound reduction in M_12_ values, indicating different pathophysiological processes may preferentially affect distinct aspects of diurnal alignment (Figure 2i).

### 2.3 Association of 24hAlign Features With Future Diseases and Mortality

**FIGURE 3:**
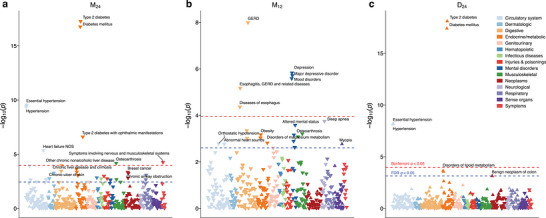
Phenome‐wide association study reveals distinct disease signatures. (a–c) Manhattan plots showing associations between 24hAlign features and 456 incident disease phenotypes across UK Biobank participants (*n* = 89,309). Each panel displays −log_10_(*p*‐values) from Cox proportional hazards models adjusted for age, sex, TDI, ethnicity, education, smoking status, alcohol consumption, healthy diet score, BMI, sleep duration, sedentary duration, MVPA duration, assessment center, and season of wear. Downward triangles indicate protective associations (HR < 1); upward triangles indicate increased risk (HR > 1). Horizontal dashed lines denote Bonferroni‐corrected significance threshold (red), and FDR *q* < 0.05 (blue).

**FIGURE 4:**
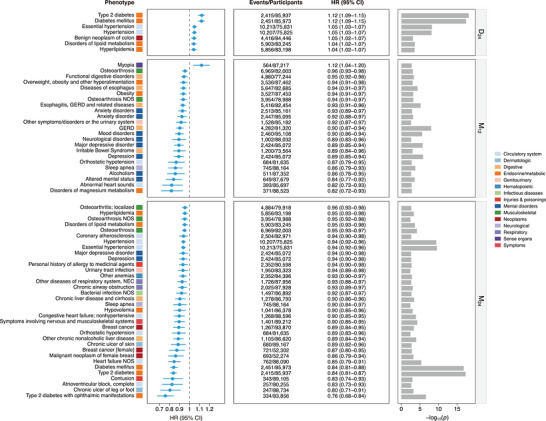
Forest plot of significant disease phenotypes in the phenome‐wide association analysis. HR and 95% CIs for incident disease phenotypes significantly associated with 24hAlign features after FDR correction. For each phenotype, the left column displays the disease name color‐coded by category, the middle column shows the number of incident events and total participants at risk, and the right column presents the forest plot with HR point estimates (dots) and 95% CIs (horizontal lines) on a logarithmic scale. Gray bars provide a visual representation of −log_10_(*p*). All models were adjusted for age, sex, TDI, ethnicity, education, smoking status, alcohol consumption, healthy diet score, BMI, sleep duration, sedentary duration, MVPA duration, assessment center, and season of wear.

We conducted a comprehensive PheWAS examining how 24hAlign features predicted future disease risk across 456 phenotypes (≥ 200 incident cases) across 15 disease groups. Over a median follow‐up period of 7.83 years, the PheWAS analyses identified 34, 23, and 7 significant associations for M_24_, M_12_, and D_24_, respectively, at Benjamini‐Hochberg FDR *q* < 0.05 after full covariate adjustment (Figure 3). Per 1 standard deviation (SD) higher M_24_ was significantly associated with lower risk of a broad range of chronic diseases, such as type 2 diabetes (hazard ratio (HR) 0.84, 95% confidence interval (CI) 0.81‐0.87), essential hypertension (HR 0.94, 95% CI 0.92‐0.96), heart failure (HR 0.85, 95% CI 0.79‐0.91), NAFLD (HR 0.89, 95% CI 0.84‐0.94), depression (HR 0.94, 95% CI 0.90‐0.98), sleep apnea (HR 0.90, 95% CI 0.84‐0.97), and notably, female breast cancer (HR 0.87, 95% CI 0.80‐0.95) (Figure 4 and Table S2). The D_24_ showed primarily cardiometabolic associations in the expected direction of misalignment, with per 1 SD higher D_24_ associated with higher risk of type 2 diabetes (HR 1.12, 95% CI 1.09‐1.15), essential hypertension (HR 1.05, 95% CI 1.03‐1.07), and disorders of lipoid metabolism (HR 1.04, 95% CI 1.02‐1.07). The M_12_ revealed associations with gastrointestinal, metabolic, and mental health outcomes. Per 1 SD higher M_12_ values, indicating stronger semidiurnal patterns, significantly associated with lower risk of GERD (HR 0.90, 95% CI 0.87‐0.94), functional digestive disorders (HR 0.95, 95% CI 0.92‐0.98), irritable bowel syndrome (HR 0.89, 95% CI 0.84‐0.96), depression (HR 0.89, 95% CI 0.85‐0.94), anxiety disorder (HR 0.93, 95% CI 0.88‐0.97), obesity (HR 0.94, 95% CI 0.91‐0.97) and sleep apnea (HR 0.86, 95% CI 0.79‐0.93). We used restricted cubic spline models to evaluate the non‐linear associations between 24hAlign features and disease phenotypes (Figure S7). In 64 pairs of significant disease‐24hAlign feature associations, we identified 11 pairs (17.19%) of disease‐24hAlign feature associations that displayed a significant non‐linear association at *p*‐value for non‐linearity < 0.05. In addition, all of the restricted cubic spline models achieved *p*‐values for overall < 0.05.

We next examined whether 24hAlign features were associated with future mortality risk. Over a median follow‐up period of 11.0 years, we observed 5,064 deaths from all causes and 723 CVD deaths. Both weaker coupling and greater phase deviation significantly predicted increased mortality risk after full covariate adjustment. Per 1 SD higher M_24_ was significantly associated with lower risk of all‐cause mortality (HR 0.93, 95% CI 0.91‐0.96) and CVD mortality (HR 0.88, 95% CI 0.82‐0.94). Similarly, per 1 SD higher D_24_ was significantly associated with higher risk of all‐cause mortality (HR 1.04, 95% CI 1.01‐1.06) and CVD mortality (HR 1.07, 95% CI 1.01‐1.13). However, the M_12_ was not associated with mortality risk (Table S3).

Several sensitivity analyses were conducted to evaluate the robustness of the primary findings. Results were largely consistent when further adjusted for shift work status in the PheWAS analyses; further adjusted for average acceleration and temperature from the acceleration data, and medication use in the PheWAS analyses; or mutually adjusted each 24hAlign feature (for example, in the PheWAS analysis of M_24_, we additionally adjusted for M_12_ and D_24_) in the PheWAS analyses (Table S4‐S6 and Figure S8); Results were consistent when excluded participants deceased within 1 year after start date of wearing the accelerometer in the mortality analyses (Table S7).

To quantify the potential public health implications of diurnal misalignment as reflected by our parameters, we calculated model‐based population attributable fractions (PAFs) for disease phenotypes significantly associated with 24hAlign features (Table S8). These estimates indicate what proportion of disease burden is statistically associated with suboptimal alignment under strong causal assumptions. For type 2 diabetes, the D_24_ contributed to 11.61% of incident cases, representing 42.04 (95% CI 31.79‐52.35) attributable cases per 100 000 person‐years. The M_24_ contributed to 26.53% of type 2 diabetes cases or 96.05 (75.40‐116.96) attributable cases per 100 000 person‐years. For type 2 diabetes with ophthalmic manifestations, M_24_ exhibited an even greater impact, contributing to 40.26% of incident cases or 20.44 (12.76‐27.49) attributable cases per 100 000 person‐years. The M_12_ exhibited large impacts on gastrointestinal, metabolic, and mental health conditions, which contributed to 17.71% of GERD cases, 19.27% of depression cases, 13.76% of anxiety disorder cases, and 11.19% of obesity cases. We also calculated that 10.42% of all‐cause mortality was attributable to low M_24_ values, corresponding to 55.41 (33.05‐77.71) excess mortality per 100 000 person‐years. High D_24_ contributed to 3.27% of all‐cause mortality or 17.37 (5.01‐28.30) excess mortality per 100 000 person‐years. Low M_24_ accounted for 19.96% of CVD mortality, while high D_24_ contributed 6.53%.

### 2.4 Environmental Factors Associated With 24hAlign Features

We investigated the associations between environmental factors and 24hAlign features, identifying 359 pairs (out of 1056, 34%) of significant associations between risk factors and 24hAlign features at the Benjamini‐Hochberg FDR *q* < 0.05 level (Table S9). Self‐reported sleep phenotypes, such as having a higher frequency of habitual nap during the day was associated with lower M_24_ (*β* = −0.08, *p* = 7.51 × 10^−130^) and higher D_24_ (*β* = 0.01, *p* = 2.08 × 10^−4^), but higher M_12_ values (*β* = 0.04, *p* = 6.11 × 10^−31^); evening chronotype was associated with lower M_24_ (*β* = −0.01, *p* = 6.53 × 10^−5^) and lower M_12_ values (*β* = −0.03, *p* = 2.85 × 10^−15^) compared to participants reporting a morning preference. Higher waist‐hip ratio was associated with lower M_24_ (*β* = −0.06, *p* = 5.12 × 10^−28^), lower M_12_ values (*β* = −0.01, *p* = 3.76 × 10^−3^) and higher D_24_ (*β* = 0.06, *p* = 4.02 × 10^−28^). Mental health was associated with misalignment as well; a higher Patient Health Questionnaire‐9 (PHQ‐9) score was associated with lower M_24_ (*β* = −0.03, *p* = 1.86 × 10^−14^), and lower M_12_ values (*β* = −0.02, *p* = 1.48 × 10^−9^), but not associated with D_24_. Diabetes diagnosed by a doctor was associated with lower M_24_ (*β* = −0.03, *p* = 1.19 × 10^−23^), and higher D_24_ values (*β* = 0.03, *p* = 7.93 × 10^−26^), but not associated with M_12_. Sun exposure was associated with misalignment but with a different direction; higher time spent outdoors in summer was associated with higher M_24_ (*β* = 0.03, *p* = 1.69 × 10^−20^), but lower M_12_ values (*β* = −0.04, *p* = 6.67 × 10^−30^). Similarly, higher time spent outdoors in winter was associated with higher M_24_ (*β* = 0.02, *p* = 1.56 × 10^−6^), and lower M_12_ values (*β* = −0.01, *p* = 6.86 × 10^−4^). Higher natural environment percentage (buffer 1000m) was associated with higher M_24_ (*β* = 0.02, *p* = 1.04 × 10^−5^), but lower M_12_ values (*β* = −0.03, *p* = 3.20 × 10^−23^).

### 2.5 Technical Replication of the 24hAlign Framework

We applied the 24hAlign framework to accelerometer data from the Survey of Health, Ageing and Retirement in Europe (SHARE) cohort (*N* = 853) to assess its technical portability across an independent dataset with a differing sensor placement. Despite the distinct signal characteristics of thigh‐worn accelerometry, the framework extracted diurnal features from the raw signals without requiring modification to the core algorithm. As shown in Figure S9, the extracted features exhibited stable distributions. Specifically, the peak lag τ of cross‐correlation showed a near‐normal distribution with a median of 13.2 (Figure S9a). The 24‐h magnitude M_24_ had a median of 0.3 (Figure S9b), the 12‐h magnitude M_12_ had a median of 0.1 (Figure S9c), and the 24‐h deviation D_24_ showed a median of 1.0 (Figure S9d). These findings confirm the technical portability of the 24hAlign framework across device protocols in older European adults, but do not constitute validation of disease associations in diverse populations.

### 2.6 Robustness Analyses of 24hAlign Features

Participant‐level missingness diagnostics confirmed that data completeness was high for the majority of participants (Figure S10). We also confirmed that data completeness was high in the SHARE cohort (Figure S11). In the high‐completeness subset of UK Biobank (*N* = 62,032), PheWAS results remained well‐aligned with the primary analysis (phenotype‐level z‐statistic correlation: M_24_ = 0.91, M_12_ = 0.88, D_24_ = 0.89; all *p* < 2.2 × 10^−16^) (Figure S12). Adjustment for completeness indicators yielded z‐statistic correlations of 1.00 for all three features (Figure S13). Acceleration‐spike winsorization produced z‐statistic correlations of 1.00 for all three features (Figure S14). After additional adjustment of M_12_ models for baseline sleep‐related traits, the M_12_‐disease associations remained highly aligned with the primary analysis (z‐statistic correlation = 0.99; Figure S15). Mortality associations for M_24_ and D_24_ were preserved across these robustness analyses (Table S10).

In exploratory higher‐harmonic analyses, M_8_ and M_6_ had substantially smaller median magnitudes than M_24_ and M_12_ (M_8_ = 0.021; M_6_ = 0.018; Figure S16). M_8_ showed limited FDR‐significant associations, including mood disorders (HR 1.07, 95% CI 1.03‐1.11), carditis (HR 1.16, 95% CI 1.09‐1.24), and cerebral artery occlusion with infarction (HR 1.13, 95% CI 1.07‐1.20). M_6_ yielded no additional FDR‐significant associations (Table S11). Neither was associated with mortality (Table S12).

## 3 Discussion

This study addressed whether cross‐domain diurnal alignment between wearable‐derived physical activity and device temperature under free‐living conditions is prospectively associated with health outcomes. Analyzing approximately 90,000 UK Biobank participants with week‐long wearable recordings, we demonstrated two principal findings, supported by extensive robustness analyses. First, our novel 24hAlign features (M_24_, D_24_, and M_12_) derived from the circular cross‐correlation function captured the expected approximate antiphase relationship between activity and device temperature, with population‐level peaks at around 11.4 h. Second, alignment predicted incident disease across multiple organ systems and mortality, with M_24_ showing broad disease associations, D_24_ demonstrating focused metabolic effects, and M_12_ revealing gastrointestinal and mental health associations. Third, multiple robustness analyses, including adjustment for non‐wear completeness, acceleration‐spike winsorization, and baseline sleep‐related traits, confirmed that the primary findings were not explained by these measured sources of confounding or artifact. The technical portability of the framework was confirmed in the independent SHARE cohort, demonstrating applicability to diverse device protocols in older European adults.

We interpret 24hAlign as a wearable‐derived measure of coupling between physical activity patterns and device‐temperature fluctuations that may partly reflect aspects of diurnal behavioral‐thermoregulatory coordination. The observed alignment may arise from multiple sources, including behavioral regularity, environmental zeitgeber fidelity (e.g., adherence to light‐dark or social schedules), peripheral thermoregulatory rhythmicity, and device‐temperature measurement properties. Previous UK Biobank investigations have demonstrated that rest‐activity rhythm amplitude predicts multiple disease outcomes and that device temperature amplitude associates with metabolic and cardiovascular conditions [16, 17]. However, these studies examined each modality in isolation. Our framework advances this foundation by quantifying the cross‐domain relationship between behavioral activity and device temperature, capturing not only whether each signal is rhythmic, but whether they maintain consistent temporal relationships.

Higher M_24_ likely reflects stronger coupling between behavioral timing and device‐temperature patterns. One physiological contributor may be the role of body temperature as a zeitgeber for peripheral clocks, although our wearable measure does not directly quantify core body temperature or tissue‐level thermal rhythms. In mammals, body temperature rhythms regulated by the SCN serve as a synchronizer of peripheral tissue clocks [8, 18]. The approximately 12 h antiphase relationship observed between activity and device temperature under typical conditions is consistent with the known opposition between daytime behavioral thermogenesis and nocturnal peripheral vasodilation that facilitates heat dissipation.

The strong association between M_24_ and type 2 diabetes (HR 0.84) may reflect multiple pathophysiological mechanisms. Diabetes introduces confounding thermal dysregulation through autonomic neuropathy and vascular dysfunction, which compromise cellular thermosensing and stress response pathways independent of circadian effects [19]. Diabetic autonomic neuropathy impairs both vasoconstriction and vasodilation, potentially reducing the amplitude of distal temperature oscillations. Physical activity during the day normally amplifies body temperature rhythms, but the reduced physical capacity often accompanying diabetes attenuates these signals [20]. Thus, low M_24_ in diabetes likely reflects a complex interplay of impaired temperature regulation, autonomic dysfunction, and behavioral changes, rather than a single upstream circadian mechanism.

The semidiurnal patterns captured by M_12_ represent the 12 h harmonic component of the free‐living activity‐device‐temperature cross‐correlation. In free‐living adults, such semidiurnal structure may arise from biphasic activity, daytime napping, split activity and rest schedules, fragmented sleep‐wake behavior, or repeated morning and evening behavioral transitions. Our finding that higher M_12_ was associated with lower risk of gastrointestinal disorders, including GERD (HR 0.90) and irritable bowel syndrome (HR 0.89), and with lower risk of depression (HR 0.89) and anxiety (HR 0.93), is notable but mechanistically unresolved. Further adjustment for baseline sleep‐related traits, including chronotype, habitual napping, insomnia, and daytime dozing, did not materially alter the M_12_‐disease associations. However, baseline sleep questionnaires cannot capture concurrent day‐to‐day napping, fragmented sleep during accelerometer wear, meal timing, medication timing, or autonomic state transitions. We therefore present the M_12_ findings as hypothesis‐generating and recommend validation in studies with direct concurrent sleep, meal timing, autonomic, and temperature measurements.

Although most associations indicate that stronger activity‐device‐temperature alignment is associated with lower risk of chronic disease and mortality, some findings are more complex. High M_12_ was associated with increased risk of myopia (HR 1.12), which may reflect lifestyle patterns that increase near‐work or indoor activity rather than a direct effect of semidiurnal rhythms. Environmental factor analyses showed that greater time outdoors was associated with higher M_24_ but lower M_12_. This opposing pattern likely arises because environmental synchronizers that consolidate a unimodal day‐night pattern strengthen the 24 h coupling (higher M_24_) while suppressing semidiurnal structure (lower M_12_). Behaviors that split rest or activity into two daily bouts increase the 12 h harmonic. Methodologically, because cross‐correlations are standardized, increases in the 24 h component can occur at the expense of the 12 h component when total rhythmic variance is limited.

Exploratory analyses of higher harmonics revealed that M_8_ (the 8 h component) showed limited associations with mood disorders and selected circulatory outcomes, while M_6_ (the 6 h component) showed no additional FDR‐significant PheWAS associations. Neither M_8_ nor M_6_ was associated with mortality. These findings suggest that the 24 h and 12 h components capture the dominant outcome‐relevant structure of the activity‐device‐temperature cross‐correlation, although higher harmonics may warrant further investigation in populations with more prevalent ultradian behavioral patterns, such as shift workers and the elderly.

Our model‐based population attributable fraction analyses suggest potentially substantial population‐level burden associated with diurnal misalignment under strong causal assumptions: 26.5% of type 2 diabetes cases and 10.4% of all‐cause mortality were statistically attributable to suboptimal M_24_. These estimates should not be interpreted as direct estimates of preventable burden, but they indicate that diurnal activity‐temperature alignment may warrant evaluation in future causal and interventional studies. The identification of modifiable upstream factors, including sleep patterns, adiposity, and environmental exposures, provides potentially actionable targets.

This study offers several key strengths. The scale and comprehensive prospective clinical follow‐up of the UK Biobank enabled disease‐relevant analysis of approximately 90 000 participants with week‐long continuous recordings, comprehensive phenotyping, and extended follow‐up exceeding 11 years for mortality outcomes. Our methodological approach incorporated robust preprocessing, careful non‐wear time handling, participant‐level standardization, and multiple sensitivity analyses, ensuring technical reliability. The naturalistic setting enhances generalizability to real‐world conditions where circadian disruption typically occurs.

This study has several limitations. First, device temperature imperfectly approximates local wrist skin temperature, is not equivalent to core body temperature, and remains susceptible to ambient conditions, variable skin contact, clothing, evaporative cooling, body composition, and device‐specific noise. Although the robustness of findings across diverse resampling strategies, standardization methods, temporal resolutions, seasonality adjustments, and non‐wear completeness analyses suggests resilience to many measurement artifacts, confirmation with direct continuous skin or core body temperature sensors remains important. Second, data collection on the dominant wrist may introduce systematic biases in activity‐temperature coupling compared with standard non‐dominant wrist protocols. Third, residual confounding remains possible despite extensive covariate adjustment. Information on direct photic history and meal timing (chrononutrition) was not collected in the original datasets (UK Biobank and SHARE). Future studies utilizing other datasets that combine wearable activity, light (at the ocular level), meal‐timing, and temperature measurements are needed to distinguish internal circadian alignment from behavioral and environmental timing compliance. Fourth, the alignment features may partly capture behavioral regularity and zeitgeber fidelity rather than internal circadian‐oscillator coordination, and these contributions cannot be separated with available data. Fifth, the seven‐day recording period represents a cross‐sectional snapshot that likely does not fully capture longitudinal stability or seasonal plasticity. Sixth, the UK Biobank cohort is predominantly White and middle‐to‐older aged, and the SHARE cohort also consists of older European adults. Generalizability to non‐White ancestries, younger demographics, and diverse socioeconomic and geographic contexts remains to be established. Finally, future studies should employ quasi‐experimental designs exploiting natural experiments such as daylight saving transitions or shift schedule changes to test causal effects on alignment and intermediate biomarkers.

In summary, 24hAlign features provide a non‐invasive window into the temporal coupling between physical activity and wearable‐derived device‐temperature patterns under free‐living conditions. The prospective associations with incident disease across multiple organ systems and with all‐cause and CVD mortality, together with the robustness across multiple sensitivity analyses, support diurnal activity‐temperature alignment as a scalable wearable‐derived predictor of future health risk. Whether strengthening this alignment through optimized light exposure, timed physical activity, or behavioral rhythm consolidation reduces the burden of circadian‐sensitive diseases remains to be tested in prospective and interventional studies.

## 4 Methods

### 4.1 Study Design and Population

We analyzed UK Biobank participants who wore wrist accelerometers and had linked electronic health records, death registries, and genetic data. The UK Biobank is a prospective cohort study of approximately 500,000 adults aged 37 to 73 years recruited between 2006 and 2010 (baseline of the UK Biobank) from across the United Kingdom to complete touchscreen questionnaires, physical examinations, and biological sample collections. Between 2013 and 2015 (baseline of this study), a subset of 103,666 participants wore Axivity AX3 triaxial accelerometers (Axivity Ltd, Newcastle upon Tyne, UK) on their dominant wrist continuously for a median of 7 days [21]. The UK Biobank received ethical approval from the North West Multi‐centre Research Ethics Committee (reference 21/NW/0157), and all participants provided written informed consent. Our analyses used deidentified data under approved application 102158. We excluded participants with unreliable accelerometry recordings if they met the following conditions: (1) recordings flagged by UK Biobank as being unreliable due to unexpectedly small or large file size (field‐id: 90002); (2) those with accelerometry recordings for less than 72 h or who did not provide data for all 1‐h periods within a 24‐h cycle during the seven‐day data collection (field‐id: 90015); (3) data identified by UK Biobank as not well‐calibrated (field‐id: 90016); (4) recordings recalibrated using the previous accelerometer record from the same device worn by a different participant (field‐id: 90017); (5) data with a non‐zero count of interrupted recording periods (field‐id: 90180); (6) recordings with more than 768 data recording errors (field‐id: 90182), a threshold derived as > Q3 + 1.5 × IQR of the error‐count distribution. We then excluded participants with missing covariates. The remaining participants were included in the primary analysis. For the PheWAS of incident disease phenotypes and for the mortality analyses, participants with a prevalent diagnosis of any included disease at the time of the accelerometry assessment were excluded from the regression models.

### 4.2 Wearable Data Acquisition and Preprocessing

The Axivity AX3 devices recorded triaxial acceleration at approximately 100 Hz (range of ± 8 gravitational (*g*)) along with device temperature from an on‐device linear thermistor. The raw accelerometry data were calibrated and preprocessed using an existing pipeline [15] with our customized parameters. We calibrated acceleration data to local gravity and resampled signals to derive minute‐level activity and temperature summaries. Device temperature served as a proxy for wrist skin temperature. In addition, we leveraged seasonal repeated measurements collected for around 3,000 participants to account for seasonality across participants (Note S2). In brief, seasonality was adjusted using a linear mixed‐effects harmonic regression, where the 24Align feature was modeled as a function of sine and cosine of day‐of‐year at annual and semiannual frequencies, a linear calendar‐time trend, and a study‐period indicator, with participant‐level random intercepts. For each observation, a season‐adjusted 24Align feature was computed by subtracting the model's population‐level seasonal component (from the fixed effects) predicted for that date, yielding values comparable across dates.

In post‐processing steps, we reconstructed local time for each participant, accounting for daylight saving transitions and segmented days from midnight to midnight local time. For acceleration, we calculated the Euclidean Norm Minus One with negative values set to zero (ENMONZ) at each minute, where $\mathrm{ENMONZ}\;\left(t\right)\;=\;\max\{\sqrt{x^{2}\;\left(t\right)+y^{2}\;\left(t\right)+z^{2}\;\left(t\right)}\;-1\}$, with *x*, *y*, and *z* representing the three acceleration axes in m*g* units. We applied a logarithmic transformation *f* (*x*) =  ln(1 + *x*) to suppress extreme spikes while preserving physiological variation. For temperature, we converted raw values to degrees Celsius using *T* (^°^C)  =  (500  ×  *X*  −  2550) / 256, where *X* is the raw value in arbitrary units.

Non‐wear time was determined from the existing processing pipeline [15]. We removed non‐wear segments from both the acceleration and device temperature series and excluded days with less than 80 percent valid data. We standardized both signals within each participant by computing robust z‐scores [22]:

(1)

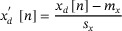



(2)

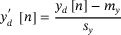

where *m* and *s* represent the median and robust standard deviation (1.4826  ×  median(|*x* − *m*|)) across all valid minutes after non‐wear removal, ensuring unit variance and preventing amplitude‐driven artifacts in cross‐participant comparisons.

### 4.3 24hAlign Feature Construction

For each participant, we computed the circular cross‐correlation between standardized acceleration and device temperature patterns averaged across valid days. The cross‐correlation function at lag τ is:

(3)

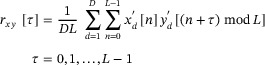

where *D* represents the number of valid days, L=1440min per day, $x_{d}^{\prime }\left[n\right]$ is the standardized acceleration at minute *n* of day *d*, and $y_{d}^{\prime }\left[n\right]$ is the standardized device temperature. The modulo operation ensures circular wrapping within each 24 h period. This correlation function captures how device temperature relates to acceleration at different time lags throughout the day.

We decomposed the cross‐correlation function using discrete Fourier transforms to isolate specific frequency components:

(4) $$R\;\left[k\right]=\sum_{\tau =0}^{L-1}e^{-i\frac{2\pi }{L}\tau k}\;r_{xy}\left[\tau \right]\;\;k=0,1,\text{…},L-1\;$$

The 24 h component (k=1) yielded the coupling magnitude:

(5) $$\begin{array}{c} \;M_{24}=\frac{2\left|R\left[1\right]\right|}{L}\;\; \end{array}$$

which ranges from 0 to 1 and quantifies the strength and regularity of activity‐temperature coupling. The phase relationship:

(6) $$\begin{array}{c} \theta_{24}\;\left(h\right)=-\arg\left(R\left[1\right]\right)\frac{24}{2\pi }\; \end{array}$$

indicates when temperature peaks occur relative to activity patterns in hours. Since physiological expectation places activity and temperature in approximate antiphase, we computed the absolute deviation:

(7) $$D_{24}=\left|\theta_{24}-12\;h\right|$$

folded to the interval [0, 12] hours. We similarly extracted the 12 h harmonic component (k=2) with a magnitude that captures semidiurnal patterns:

(8) $$\begin{array}{c} \;M_{12}=\frac{2\left|R\left[2\right]\right|}{L}\;\; \end{array}$$

We implemented quality control for derived features. We enforced numerical bounds 0 ≤ *M*_24_ ≤ 1 with small tolerance for finite‐sample deviations. We assessed reliability through bootstrap resampling across days to estimate within‐subject standard errors, recomputing features at different temporal resolutions, seasonality adjustment, and with z‐score standardization. Per harmonic *k* (cycles/day), we report two cross‐spectral summaries. Energy share is the cohort median of each participant's $w_{k}={\left|R[k]\right|}^{2}/\sum_{j\geq 1}{\left|R[j]\right|}^{2}$, i.e., the fraction of total cross‐correlation energy attributable to harmonic *k*. A significant proportion is the fraction of participants whose coupling at *k* exceeds a day‐wise circular‐shift permutation null (|*R*[*k*]| test with Benjamini‐Hochberg FDR *q* < 0.05). Please refer to Note S1 for a detailed description.

### 4.4 Clinical Phenotyping and Mortality

We mapped hospital inpatient records to phenotypes (phecodes) [23] using their corresponding International Classification of Diseases (ICD) codes with the Phecode Map v1.2b1 and v1.2 for both ICD‐10 and ICD‐9 codes. We included phecodes with at least 200 cases and defined case status based on any recorded diagnosis after the start of the first accelerometry assessment. We calculated follow‐up time from the start of accelerometer wear to the occurrence of death or the censoring date of hospital inpatient data (31 October 2022 for England, 31 August 2022 for Scotland, and 31 May 2022 for Wales), whichever came first. For each phenotype analyzed, participants with similar or related conditions were excluded according to exclusion criteria provided by the Phecode Map. For instance, to define cases of essential hypertension, participants with ICD codes mapping to phecode 401.1 (essential hypertension) were included. For the control group, participants without phecode 401 (hypertension) were included, excluding related conditions and potential undiagnosed cases, such as hypertensive heart disease (phecode 401.21), hypertensive chronic kidney disease (phecode 401.22), and other hypertensive complications (phecode 401.3). Importantly, exclusions were phenotype‐specific, meaning participants excluded from one phecode analysis remained eligible for other analyses unless they met those specific exclusion criteria as well.

For mortality outcomes, we identified date and cause of death through linkage to the National Health Service (NHS) Information Center (England and Wales) and the NHS Central Register (Scotland). CVD mortality was defined by ICD‐10 codes: I20‐I25, I46, I50, and I60‐I64. We calculated follow‐up time from the start of accelerometer wear to death or the censoring date of death registry linkage (31 May 2024 for England and Wales, and 31 December 2023 for Scotland).

Of the 89,309 participants meeting the inclusion criteria for this study, we identified 456 phecodes with a median follow‐up of 7.83 years. For mortality outcomes, we identified a median follow‐up of 11.0 years.

### 4.5 Covariates

Covariates included age, sex (male, female), Townsend deprivation index (TDI), ethnicity (White, non‐White), education (less than high school, high school or equivalent, college or above), smoking status (never, previous, current), alcohol consumption (none currently, up to twice per week, three or more times per week), healthy diet score, body mass index (BMI), accelerometer‐measured sleep duration, sedentary duration, and moderate‐to‐vigorous physical activity (MVPA) duration, assessment center (England, Scotland, Wales), and season of wear (spring: March‐May; summer: June‐August; autumn: September‐November; winter: December‐February). Age was calculated from the date of birth and the start date of the first accelerometer assessment. Data on sex were acquired from the central registry at recruitment. TDI was collected via questionnaires at initial assessment, which represents the level of deprivation on the basis of postcodes; it was derived from aggregated data of unemployment, car ownership, house ownership, and household overcrowding, with higher scores indicating higher deprivation [24]. Healthy diet score (ranging 0–7) was calculated by assigning 1 point for a healthy frequency and 0 points for an unhealthy frequency of consumption of fruits, vegetables, fish, processed meat, unprocessed red meat, whole grains, and refined grains, with higher scores indicating better diet quality [25, 26]. Sleep duration, sedentary duration, and MVPA duration were quantified from accelerometer data as hours per day. See Note S3 for detailed covariate definitions.

Baseline refers to the start date of the first accelerometer assessment (2013‐2015). In the UK Biobank, participants may attend the initial baseline assessment (instance 0, 2006–2010), the first repeat assessment (instance 1, 2012–2013), the imaging visit (instance 2, from 2014), and the first repeat imaging visit (instance 3, from 2019). For all covariates, we used the measurement collected closest in time to the accelerometer baseline, except that age (as defined above), sex, assessment center, ethnicity, TDI, and accelerometer‐derived sleep duration, sedentary duration, and MVPA duration.

### 4.6 Statistical Analyses

For the PheWAS analyses, we used the Cox proportional hazards model to test associations between 24hAlign features and each phecode. We modeled M_24_, D_24_, and M_12_ as exposures individually, with adjustment to the full covariate set, including age, sex, TDI, ethnicity, education, smoking status, alcohol consumption, healthy diet score, BMI, sleep duration, sedentary duration, MVPA duration, assessment center, and season of wear. All 24hAlign features (M_24_, D_24_, M_12_) were standardized (z‐scored; mean 0, SD 1) before modeling. Unless stated otherwise, HRs and 95% CIs are interpreted per 1 SD higher value of the feature. For M_24_ and M_12_, higher values indicate stronger alignment; for D_24_, higher values indicate greater misalignment. We excluded participants who were diagnosed with relevant diseases within 1 year after the start date of wearing the accelerometer to address potential reverse causality. We explored nonlinear relationships using restricted cubic splines and reported associations per SD change. We controlled FDR at 0.05 using the Benjamini‐Hochberg procedure [27] across all phecodes tested. The Bonferroni method was also used for multiple testing correction, with Bonferroni‐corrected *p* < 0.05 as the significance level. For mortality analyses, we employed Cox proportional hazards models with age as the time scale. We verified proportional hazards assumptions using Schoenfeld residuals and allowed time‐varying effects where necessary [28]. We employed restricted cubic spline models [29] to examine potential nonlinear associations between disease phenotypes. Potential nonlinearity was assessed by Wald tests. Four knots were used, i.e., at the fifth, 35th, 65th, and 95th percentiles of each 24hAlign feature distribution, and the reference value was set at the median of each 24hAlign feature.

We calculated PAFs to estimate the proportion of disease burden potentially preventable through optimization of 24hAlign features. For each 24hAlign features, we defined reference categories based on the distribution in the healthy population. For M_24_ and M_12_, where higher values indicate better alignment, we used the 95th percentile as the reference category representing optimal coupling strength. For D_24_, where lower values indicate better alignment, we used the fifth percentile as the reference category representing minimal phase deviation. We computed PAFs using the standard formula [30]:

(9) $$\mathrm{PAF}=\frac{P\left(\mathrm{exposed}\right)\times \left(\mathrm{HR}-1\right)}{1+P\left(\mathrm{exposed}\right)\times \left(\mathrm{HR}-1\right)}$$

where P(exposed) represents the proportion of the population below the 95th percentile for M_24_ and M_12_ or above the fifth percentile for D_24_, and HR represents the hazard ratio comparing the exposed group to the reference category obtained from Cox proportional hazards models with full covariate adjustment. To translate PAFs into absolute numbers, we calculated attributable cases per 100 000 person‐years by multiplying the PAF by the observed incidence density for each outcome. We computed 95% CIs for PAFs using the bootstrap resampling.

To identify upstream environmental factors of 24hAlign features, we regressed 24hAlign features on factors including cognitive function (*n* = 20), early‐life risks (*n* = 10), local environment (*n* = 29), lifestyle factors (*n* = 109), mental health (*n* = 91), and physical health (*n* = 93). Linear regression models were controlling for age, sex, TDI, ethnicity, education, smoking status, alcohol consumption, healthy diet score, BMI, sleep duration, sedentary duration, MVPA duration, assessment center, and season of wear. Please refer to Table S13 for variable description. We reported the standardized *β* coefficient and 95% CI.

We conducted several sensitivity analyses. First, we additionally adjusted the PheWAS models for shift‐work status. Second, we further adjusted the PheWAS models for average temperature and acceleration from the accelerometer, and medication use. Third, the M_24_, M_12_, and D_24_ were mutually adjusted in the PheWAS models to assess whether the associations were attributable to any of these three features. Finally, to address potential reverse causality, we excluded participants who died within 1 year of the accelerometer wear start date.

We conducted several additional robustness analyses. First, to evaluate whether non‐wear periods or temporal discontinuities biased the cross‐correlation estimates, we quantified participant‐level missingness and lag‐wise effective sample sizes among the final analytic sample (*N* = 89,309). For each participant, we calculated the overall valid‐minute proportion, valid‐minute proportions during nighttime (22:00‐06:00), dawn transition (04:00‐09:00), and dusk transition (17:00‐22:00), the longest continuous missing segment, the longest nighttime missing segment, the number of nights with at least 60 consecutive missing minutes, and the ratio of the minimum to maximum number of valid activity‐temperature pairs across cross‐correlation lags. We repeated the PheWAS and mortality analyses in a high‐completeness subset defined by nighttime valid proportion ≥ 90%, longest nighttime gap ≤ 60 min, and lag‐wise effective‐sample ratio ≥ 90%. We also repeated all analyses with additional covariate adjustment for the above completeness indicators. Likewise, we calculated the above completeness indicators in the SHARE cohort. Second, to assess the potential influence of abrupt acceleration spikes (e.g., from alarm‐clock‐driven awakenings), we recomputed 24hAlign features after winsorizing extreme minute‐level acceleration values at the 99th percentile applied across the full 24‐h day. The PheWAS and mortality analyses were then repeated using the winsorized features. Third, for M_12_ analyses specifically, we additionally adjusted the PheWAS models for available baseline sleep‐related traits, including chronotype, habitual napping frequency, insomnia symptom frequency, and daytime dozing frequency. Fourth, as exploratory analyses, we extracted the *k* = 3 and *k* = 4 harmonic magnitudes (M_8_ and M_6_) from the cross‐spectral decomposition and tested their associations with all 456 phenotypes and with mortality using the same Cox proportional hazards framework and covariate adjustment as the primary analyses. The main analyses were performed using R v4.3.1. All *p*‐values were two‐sided.

### 4.7 Replication of the 24Align Framework

To rigorously test the generalizability of our framework, we utilized the accelerometer sub‐study from Wave 8 of the SHARE. SHARE is a multidisciplinary and cross‐national panel database covering individuals aged 50 years and older across Europe [31]. The data collection for this wave took place largely between 2019 and 2020 across 10 European countries. Participants in the accelerometer study were instructed to wear a triaxial accelerometer (Axivity AX3, Axivity Ltd, Newcastle upon Tyne, UK) on a belt secured on the upper thigh for eight consecutive days [32]. While the device model is identical to that used in the UK Biobank, the thigh‐worn placement introduces distinct signal characteristics and movement artifacts compared to the wrist‐worn protocol in the primary dataset.

## Author Contributions

X.T. and Z.C. were involved in the conception, design, and conduct of the study, and the analysis and interpretation of the results. H.C. wrote the first draft of the manuscript, and all authors edited, reviewed, and approved the final version of the manuscript. H.C. and J.W. analysed the data and interpreted the results. J.C., C.B., and A.T. provided critical revision of the manuscript for important intellectual content. X.T. and H.C. directly accessed and verified the underlying data reported in the manuscript. X.T. had full access to all the data in the study and takes responsibility for the integrity of the data and the accuracy of the data analysis.

## Conflicts of Interest

The authors declare no conflicts of interest.

## Supporting information




**Supporting File 1**: advs76217‐sup‐0001‐SuppMat.docx.

**Supporting File 2**: advs76217‐sup‐0002‐Tables.xlsx.

## Data Availability

UK Biobank data are available to bona fide researchers via application at https://www.ukbiobank.ac.uk/. This analysis used the UK Biobank application number 102158. The SHARE Wave 8 Raw Accelerometer Data are available for research purposes via the SHARE Research Data Center (https://releases.sharedataportal.eu/). Access is granted to registered researchers upon acceptance of the SHARE Conditions of Use, and access to raw accelerometer data is subject to additional restrictions to protect participant privacy. Custom analysis scripts for the 24hAlign framework are publicly available at https://github.com/sakuramodokich/acceleration_temp.
