# Supplementary material for: Wearable‐Derived Diurnal Alignment Between Physical Activity and Device Temperature Predicts Future Disease and Mortality Risk
Source: Adv Sci (Weinh). 2026 Jun 19;13(52):e76217. doi: 10.1002/advs.76217 (PMC13337042; doi:10.1002/advs.76217)
Supplement: Supplementary file 1 [file ADVS-13-e76217-s001.docx]

**Supporting information for: Wearable-derived diurnal alignment between physical activity and device temperature predicts future disease and mortality risk**

*Han Chen, Jiahe Wei, Jonathan Cedernaes, Christian Benedict, Athanasios Tsanas, Zhi Cao^*^, Xiao Tan^*^*

^*^Correspondence to Zhi Cao and Xiao Tan: [caozhi@zju.edu.cn](mailto:caozhi@zju.edu.cn); [xiao.tan@zju.edu.cn](mailto:xiao.tan@zju.edu.cn)

**Contents**

[Supplementary Notes on Methods	4](#_Toc230785710)

[Supplementary Note 1.	Description of circular cross-correlation and spectral decomposition	4](#_Toc230785711)

[Supplementary Note 2.	Description of population and participant-specific seasonality	8](#_Toc230785712)

[Supplementary Note 3.	Description of covariates	12](#_Toc230785713)

[Supplementary Note 4.	Description of shift workers and medication use	13](#_Toc230785714)

[Supplementary Figure 1 Flow chart for accelerometer data quality control	15](#_Toc230785715)

[Supplementary Figure 2 Distribution of alignment features	16](#_Toc230785716)

[Supplementary Figure 3 Comparisons between features derived from bootstrap and the original method	17](#_Toc230785717)

[Supplementary Figure 4 Comparisons between recomputing features and the original features	18](#_Toc230785718)

[Supplementary Figure 5 Comparisons between features removing seasonality and the original features	19](#_Toc230785719)

[Supplementary Figure 6 Cross-spectral decomposition of circular cross-correlation across diurnal harmonics	20](#_Toc230785720)

[Supplementary Figure 7 Potential nonlinear associations between alignment features and disease phenotypes	22](#_Toc230785721)

[Supplementary Figure 8 Comparisons between sensitivity analyses and the original PheWAS analyses	23](#_Toc230785722)

[Supplementary Figure 9 Technical validation of alignment features in the SHARE cohort	24](#_Toc230785723)

[Supplementary Figure 10 Distribution of participant-level completeness indicators in the UK Biobank	25](#_Toc230785724)

[Supplementary Figure 11 Distribution of participant-level completeness indicators in the SHARE	26](#_Toc230785725)

[Supplementary Figure 12 Comparisons between high-completeness subset and the original PheWAS associations	27](#_Toc230785726)

[Supplementary Figure 13 Comparisons between completeness-indicators adjustment and the original PheWAS associations	28](#_Toc230785727)

[Supplementary Figure 14 Comparisons between acceleration-spike winsorization and the original PheWAS associations	29](#_Toc230785728)

[Supplementary Figure 15 Comparisons between baseline sleep-related traits adjustment and the original PheWAS associations	30](#_Toc230785729)

[Supplementary Figure 16 Distribution of higher harmonic components	31](#_Toc230785730)

[References	32](#_Toc230785731)

Supplementary Notes on Methods

Supplementary Note 1. Desc**ription of circula**r cross-correlation and spectral decomposition

This section expands the main-text description by deriving the indices used to quantify the diurnal coupling between activity and device temperature. We formalize the circular cross‑correlation across days, its discrete Fourier transform (DFT), and the extraction and interpretation of the 24-h and 12-h harmonic components.

1.1 **Notation and preprocessing**

Let a civil day have duration $T = 24 h$ discretized into $L$ equally spaced samples (e.g., $L = 1440$ for 1 min resolution), indexed by $n = 0, \ldots, L-1$. For participant‑level analysis with $D$ valid days ($d = 1, \ldots, D$), let $x_{d}\left[ n \right]$ denote the activity series and $y_{d}\left[ n \right]$ denote device temperature at minute $n$ of day $d$. After excluding non-wear periods, we apply spike suppression to activity via $f\left( x \right)=\ln\left( 1+\max\left( 0,x \right) \right)$ and perform participant-level robust z-scoring across all valid samples pooled over days:

$$x_{d}^{'}\left[ n \right]=\frac{x_{d}\left[ n \right]-m_{x}}{s_{x}},\quad\quad y_{d}^{'}\left[ n \right]=\frac{y_{d}\left[ n \right]-m_{y}}{s_{y}}$$

$$m_{x}=\mathrm{median}\left( x \right),\quad\quad s_{x}=1.4826\times\mathrm{median}\left( \left| x-m_{x} \right| \right)$$

yielding standardized series $x_{d}^{'}\left[ n \right],y_{d}^{'}\left[ n \right]$ with approximately unit variance and reduced sensitivity to amplitude-driven between-subject differences.

1.2 **Day-averaged circular cross‑correlation**

Define the day‑averaged circular cross-correlation between the standardized series as

$$\begin{aligned} r_{xy}\left[ \tau\right]=\frac{1}{DL}\sum_{d=1}^{D} \sum_{n=0}^{L-1} x_{d}^{'}\left[ n \right]y_{d}^{'}\left[ \left( n+\tau\right) \mathrm{mod} L \right] \tau=0,1,\ldots,L-1\#\left( 1.1 \right) \end{aligned}$$

With z-scored inputs, $r_{xy}\left[ \tau\right] \in\mathbb{R}$, is periodic with period $L$. With mean-variance standardization, $r_{xy}\left[ 0 \right]$ (defined below) equals the pooled zero-lag Pearson correlation. Under robust z-scoring, $r_{xy}\left[ 0 \right]$ equals the zero-lag inner product under the chosen scaling and is typically very close to (but not exactly) the Pearson correlation. Intuitively, $r_{xy}\left[ \tau\right]$ quantifies how well temperature matches activity when temperature is circularly delayed by $\tau$ minutes within the day.

1.3 **Discrete Fourier transform of the cross‑correlation**

Let the length-$L$ DFT and its inverse be

$$\begin{aligned} X_{d}\left[ k \right]=\sum_{n=0}^{L-1} e^{-i\frac{2\pi}{L}nk}x_{d}^{'}\left[ n \right],\quad\quad Y_{d}\left[ k \right] = \sum_{n=0}^{L-1} e^{-i\frac{2\pi}{L}nk}y_{d}^{'}\left[ n \right]\#\left( 1.2 \right) \end{aligned}$$

$$x_{d}^{'}\left[ n \right]=\frac{1}{L}\sum_{k=0}^{L-1} e^{+i\frac{2\pi}{L}nk}X_{d}\left[ k \right],\quad\quad y_{d}^{'}\left[ n \right] = \frac{1}{L}\sum_{k=0}^{L-1} e^{+i\frac{2\pi}{L}nk}Y_{d}\left[ k \right]$$

Define the DFT of $r_{xy}$ with respect to lag $\tau$:

$$\begin{aligned} R\left[ k \right]=\sum_{\tau=0}^{L-1} e^{-i\frac{2\pi}{L}\tau k}r_{xy}\left[ \tau\right] k=0,1,\ldots,L-1\#\left( 1.3 \right) \end{aligned}$$

with inverse

$$r_{xy}\left[ \tau\right]=\frac{1}{L}\sum_{k=0}^{L-1} e^{+i\frac{2\pi}{L}\tau k}R\left[ k \right] \tau=0,1,\ldots,L-1$$

Because $r_{xy}\left[ \tau\right]\in\mathbb{R}$, $R\left[ L-k \right]=\overline{R\left[ k \right]}$ (conjugate symmetry).

Under the definitions in (1.1)-(1.3), we have

$$\begin{aligned} R\left[ k \right] = \frac{1}{DL}\sum_{d=1}^{D} \overline{X_{d}\left[ k \right]} Y_{d}\left[ k \right],\quad\quad k=0,1,\ldots,L-1\#\left( 1.4 \right) \end{aligned}$$

Thus $R\left[ k \right]$ is the day-averaged cross-periodogram at harmonic $k$ up to the constant factor $1/L$.

**Proof.**

Substitute (1.1) into (1.3) and change variables $m=\left( n+\tau\right) \mathrm{mod} L$:

$$R\left[ k \right]=\frac{1}{DL}\sum_{d=1}^{D} \sum_{n=0}^{L-1} x_{d}^{'}\left[ n \right]\sum_{\tau=0}^{L-1} y_{d}^{'}\left[ \left( n+\tau\right) \mathrm{mod} L \right]e^{-i\frac{2\pi}{L}\tau k}$$

$$=\frac{1}{DL}\sum_{d=1}^{D} \sum_{n=0}^{L-1} x_{d}^{'}\left[ n \right]e^{+i\frac{2\pi}{L}nk}\sum_{m=0}^{L-1} y_{d}^{'}\left[ m \right]e^{-i\frac{2\pi}{L}mk}$$

$$=\frac{1}{DL}\sum_{d=1}^{D} \left( \sum_{n=0}^{L-1} x_{d}^{'}\left[ n \right]e^{+i\frac{2\pi}{L}nk} \right) \left( \sum_{m=0}^{L-1} y_{d}^{'}\left[ m \right]e^{-i\frac{2\pi}{L}mk} \right)$$

$$=\frac{1}{DL}\sum_{d=1}^{D} \overline{X_{d}\left[ k \right]}Y_{d}\left[ k \right]$$

since $\overline{X_{d}\left[ k \right]}=\sum_{n} x_{d}^{'}\left[ n \right]e^{+i\frac{2\pi}{L}nk}$ by (1.2).

**Interpretation.**

Equation (1.4) shows that:

(i) $\left| R\left[ k \right] \right|$ measures cross-domain coupling magnitude at harmonic $k$;
(ii) $\arg\left( R[k] \right)$ is the phase of $y$ relative to $x$ at harmonic $k$.

For diurnal analysis, $k=1$ is the $24 h$ fundamental, $k=2$ the $12 h$ semidiurnal, etc.

1.4 **Pure sinusoid case (*k* = 1) and exact solution**

Assume the dominant 24‑h components of $x$ and $y$ are cosines with unit variance after standardization:

$$x^{'}\left[ n \right]=\sqrt{2}\cos\left( \omega n+\alpha\right)$$

$$y^{'}\left[ n \right]=\sqrt{2}\cos\left( \omega n+\beta\right)$$

$$\omega=\frac{2\pi}{L}$$

The circular cross‑correlation over a full day is:

$$r_{xy}\left[ \tau\right]=\mathrm{mea}n_{n}\left( x^{'}\left[ n \right]y^{'}\left[ n+\tau\right] \right)=2\mathrm{mea}n_{n}\left( \cos\left( \omega n+\alpha\right)\cos\left( \omega\left( n+\tau\right)+\beta\right) \right)=\cos\left( \omega\tau+\left( \beta-\alpha\right) \right)$$

where $\mathrm{mea}n_{n}$ denotes the average over $n = 0, \ldots, L-1$. The cross-term at $2\omega$ averages to zero over an integer number of cycles ($L \geq3$), which can be shown using the geometric series of complex exponentials.

Taking the DFT at $k = 1$:

$$R\left[ 1 \right]=\sum_{\tau=0}^{L-1} e^{-i\omega\tau}\cos\left( \omega\tau+\varphi\right)$$

with $\varphi= \beta- \alpha$. Using Euler’s identity and the vanishing of the geometric sum at $\pm2\omega$,

$$R\left[ 1 \right]=\frac{L}{2}e^{i\varphi}$$

Define the complex 24‑h coefficient $P_{1}:=\left( 2/L \right)R\left[ 1 \right]=e^{i\varphi}$. Then magnitude:

$$M_{24}=\left| P_{1} \right|=\frac{2\left| R\left[ 1 \right] \right|}{L}=1$$

Phase:

$$\varphi=\arg\left( P_{1} \right)=\arg\left( R\left[ 1 \right] \right)=\beta- \alpha$$

With the sign convention, the phase offset is:

$$\theta_{24}=-\varphi=\alpha- \beta$$

and in hours:

$$\theta_{24}\left( \mathrm{hours} \right)=-\arg\left( R\left[ 1 \right] \right)\frac{24}{2\pi}$$

Thus, for ideal sinusoids, $r_{xy}\left[ \tau\right]$ is exactly a 24-h cosine with amplitude 1 and phase $\beta- \alpha$.

1.5 **General (non-sinusoidal) case and definitions**

Because $e^{-i\frac{2\pi}{L}\tau k}$ form an orthogonal basis, the DFT coefficient at $k = 1$ is the complex projection of $r_{xy}\left[ \tau\right]$ onto the 24-h basis:

$$\frac{2}{L}R\left[ 1 \right]=A_{1}e^{i\varphi_{1}}$$

The 24‑h component of $r_{xy}\left[ \tau\right]$ can be written as

$$r_{xy}\left[ \tau\right]\approx A_{1}\cos\left( \omega\tau+\varphi_{1} \right)+\left( constant+higher harmonics \right)$$

Since $r_{xy}\left[ \tau\right]\in\left[ -1,1 \right]$, its projection amplitude cannot exceed 1; hence $0\leq M_{24}\leq1$ (allowing small finite-sample deviations in practice).

1.6 **Feature definitions (24h and 12h)**

For harmonic $k\geq1$, define

$$M_{k}:=\frac{2\left| R\left[ k \right] \right|}{L}\in\left[ 0,1 \right]$$

$$\theta_{k} \left( \text{hours} \right):=-\arg\left( R\left[ k \right] \right) \frac{24}{2\pi k} \in\left( -12,12 \right]$$

$$w_{k}:=\frac{\left| R\left[ k \right] \right|^{2}}{\sum_{j\in\mathcal{K}} \left| R\left[ j \right] \right|^{2}}\quad\left( energy share;see below \right)$$

We set $M_{24}:=M_{1}$, $\theta_{24}:=\theta_{1}$, and $M_{12}:=M_{2}$. Physiologically, activity and temperature are expected to be near antiphase; we therefore define the absolute deviation from antiphase

$$D_{24} = \left| \text{wra}\text{p}_{\text{±}\text{12}}\left( \theta_{24}-12\text{ h} \right) \right| \in\left[ 0,12 \right]$$

where $\text{wra}\text{p}_{\text{±}\text{12}}\left( h \right)=\left( \left( h+12 \right) \mathrm{mod} 24 \right)-12$ folds hours to $\left( -12,12 \right]$.

1.7 **Energy decomposition via Parseval**

By Parseval’s theorem for the DFT pair $\left( r_{xy}\left[ \tau\right],R\left[ k \right] \right)$,

$$\sum_{\tau=0}^{L-1} r_{xy}\left[ \tau\right]^{2} = \frac{1}{L}\sum_{k=0}^{L-1} \left| R\left[ k \right] \right|^{2}$$

Let the positive-harmonic index set be

$$\mathcal{K=}\{1,2,\ldots,K_{\max}\},\quad\quad K_{\max}:=\left\lfloor\frac{L - 1}{2} \right\rfloor$$

i.e., we exclude DC ($k = 0$) and, if $L$ is even, the Nyquist term ($k = L/2$). Because $R\left[ L-k \right]=\overline{R\left[ k \right]}$, negative or positive frequency pairs contribute equally to the total energy. We therefore define the normalized positive-frequency energy shares

$$w_{k} = \frac{\left| R\left[ k \right] \right|^{2}}{\sum_{j\in\mathcal{K}} \left| R\left[ j \right] \right|^{2}},\quad\quad\sum_{k\in\mathcal{K}} w_{k}=1$$

The quantity $w_{k}$ represents the share of cross-correlation “energy” attributable to harmonic $k$ (within positive frequencies).

1.8 **Subject-level permutation test for harmonic significance**

To assess whether coupling at harmonic $k$ exceeds chance, we use a day-wise circular-shift permutation that preserves within-day marginal spectra while destroying cross-day phase alignment between modalities:

1. For each day $d$, draw a shift $s_{d}\sim\text{Unif}\{0,\ldots,L-1\}$ and define $y_{d}^{\left( \text{perm} \right)}\left[ n \right]=y_{d}^{'}\left[ \left( n+s_{d} \right) \mathrm{mod} L \right]$.
2. Compute $R^{\left( \text{perm} \right)}\left[ k \right]$ from $\{x_{d}^{'}\left[ n \right],y_{d}^{\left( \text{perm} \right)}\left[ n \right]\}$.
3. Use $T_{k}=\left| R\left[ k \right] \right|$ as the test statistic. With $B$ permutations, the one-sided *p*-value is

$$p_{k}^{\text{perm}} = \frac{1+\sum_{b=1}^{B} \boldsymbol{1}\left\{ \left| R^{\left( \text{perm},b \right)}\left[ k \right] \right|\geq\left| R\left[ k \right] \right| \right\}}{B+1}$$

4. Apply Benjamini-Hochberg FDR across tested harmonics $\mathcal{K}$ to obtain $q_{k}$.
   1.9 **Population-level harmonic selection**

Across a cohort, we summarize per-harmonic quantities by medians: $\mathrm{med} w_{k}$ and $\Pr\left( q_{k}\leq\alpha\right)$ $(\alpha=0.05$). In our data, $k=1$ and $k=2$ together explain 97.4% of the cross-correlation energy with high significance rates.

1.10 **Peak lag hour of the cross-correlation**

We defined the peak lag as the circular lag within the 24‑h cycle at which the day‑averaged circular cross‑correlation attains its maximum:

$$\tau_{\mathrm{peak}}=\underset{\tau\in0,\ldots,L-1}{\text{argmax}}r_{xy}\left[ \tau\right]$$

We report the peak lag in hours as:

$$\tau_{\mathrm{peak}}\left( h \right)=\frac{24}{L}\tau_{\mathrm{peak}}$$

optionally wrapped to the symmetric interval $\left( -12,12 \right]$ via:

$$\tau_{\mathrm{peak}}\left( h \right)_{\pm12}=\left( \left( \tau_{\mathrm{peak}}\left( h \right)+12 \right) \mathrm{mod} 24 \right)-12$$

Because $r_{xy}\left[ \tau\right]$ is discrete, we optionally refine the estimate by quadratic (parabolic) interpolation around the discrete maximum using its two circular neighbors. Let $\hat{\tau}$ be the maximizing index and let $r_{-1}$, $r_{0}$, $r_{+1}$ be the values at $\hat{\tau}-1$, $\hat{\tau}$, $\hat{\tau}+1$ (indices taken modulo $L$). The sub‑sample offset is:

$$\delta=0.5\frac{r_{-1}-r_{+1}}{r_{-1}-2r_{0}+r_{+1}}$$

clipped to $\left[ -0.5,0.5 \right]$ for numerical stability. The refined peak lag is:

$$\tau_{\mathrm{pea}k^{*}}=\left( \hat{\tau}+\delta\right) \mathrm{mod} L$$

and in hours as:

$$\tau_{\mathrm{pea}k^{*}}\left( h \right)=\frac{24}{L}\tau_{\mathrm{pea}k^{*}}$$

$\tau_{\mathrm{peak}}$ summarizes the timing that maximizes the full (possibly non-sinusoidal) $r_{xy}\left[ \tau\right]$. It can differ from the 24-h phase $\theta_{24}$ derived from $R\left[ 1 \right]$. They coincide when $r_{xy}\left[ \tau\right]$ is well approximated by a single 24-h cosine.

1.11 **Quality control**

We enforce $0\leq M_{24},M_{12}\leq1$ with a small tolerance to accommodate finite‑sample and missing‑data effects. We use day‑level bootstrap resampling (resample the $D$ valid days with replacement) to obtain median estimates for $M_{24}$, $\theta_{24}$, and $M_{12}$. We recompute features under alternative temporal resolutions (i.e., different $L$) and confirm stability under z‑score standardization. The formulation is resolution‑agnostic; only the mapping from $\arg\left( R\left[ k \right] \right)$ to hours depends on $L$ via the factor $T/k = 24 h/k$.

Supplementary Note 2. Description of population and participant-specific seasonality

This section aims to (1) models and removes seasonality across all participants, (2) allows participant-specific seasonal deviations, and (3) decomposes the variance into interpretable components: between-person baseline, seasonality (population-level and person-specific), and residual.

2.1 **Model and notation**

Let $i=1,\ldots,N$ index participants and $j=1,\ldots,n_{i}$ index repeated measurements. Define the day-of-year angle:

$$\theta_{ij}=\frac{2\pi}{365.25} \text{doy}_{ij}\in\left[ 0,2\pi\right)$$

Let $x_{ij}\in\mathbb{R}^{p}$ denote non-seasonal covariates (e.g., study period indicator, slow linear trend), and let

$$\Phi_{M}\left( \theta\right)=\left( \sin\theta, \cos\theta, \sin\left( 2\theta\right), \cos\left( 2\theta\right), \ldots, \sin\left( M\theta\right), \cos\left( M\theta\right) \right)^{\top}\in\mathbb{R}^{2M}$$

We consider the following linear mixed model (LMM) for the alignment feature outcome $Y_{ij}$:

$$\begin{aligned} Y_{ij} = \beta_{0} + x_{ij}^{\top}\gamma+ \underset{p\text{opulation (fixed) seasonality}}{\underbrace{\beta_{\phi}^{\top}\Phi_{M}\left( \theta_{ij} \right)}} + \underset{\mathrm{baseline}}{\underbrace{b_{0i}}} + \underset{participant-specific seasonality}{\underbrace{b_{i,\phi}^{\top}\Phi_{R}\left( \theta_{ij} \right)}} + \varepsilon_{ij}\# \left( 2.1 \right) \end{aligned}$$

where:

- $\beta_{0}\in\mathbb{R}$ and $\gamma\in\mathbb{R}^{p}$ are non-seasonal fixed effects.
- $\beta_{\phi}\in\mathbb{R}^{2M}$ are fixed seasonal coefficients.
- $b_{0i}\in\mathbb{R}$ is the participant-specific random intercept.
- $b_{i,\phi}\in\mathbb{R}^{2R}$ are participant-specific random seasonal coefficients for the first $R\leq M$ harmonics.
- $\varepsilon_{ij}\sim\mathcal{N}\left( 0,\sigma^{2} \right)$ are i.i.d. residuals.

Random effects are assumed to satisfy

$$\left( \begin{matrix} b_{0i} \\ b_{i,\phi} \end{matrix} \right) \sim\mathcal{N}\left( 0,G \right), \varepsilon_{ij} ⟂ \left( b_{0i},b_{i,\phi} \right), \text{and} \left( b_{0i},b_{i,\phi} \right) ⟂ \theta_{ij}$$

We also assume that non-seasonal regressors $x_{ij}$ are either treated as fixed or we consider the variance of the seasonality-adjusted outcome $Y_{ij}-\beta_{0}-x_{ij}^{\top}\gamma$.

**Goal.** Decompose the unconditional variance (across participants and the annual cycle) into interpretable components: baseline (between-person), population seasonality, person-specific seasonality, and residual.

2.2 **Trigonometric orthogonality**

Let $\mu$ denote the distribution of $\theta$. In the idealized case of a uniform calendar over the year,

$$\theta\sim Unif\left[ 0,2\pi\right)$$

we have the well-known orthogonality identities for integers $m,n\geq1$:

$$\begin{aligned} \mathbb{E}\left[ \sin\left( m\theta\right) \right]=0, \mathbb{E}\left[ \cos\left( m\theta\right) \right]=0, \# (2.2) \\ \mathbb{E}\left[ \sin\left( m\theta\right)\sin\left( n\theta\right) \right]=\left\{ \begin{matrix} \frac{1}{2}, & m=n, \\ 0, & m\neq n, \end{matrix} \right. \mathbb{E}\left[ \cos\left( m\theta\right)\cos\left( n\theta\right) \right]=\left\{ \begin{matrix} \frac{1}{2}, & m=n, \\ 0, & m\neq n, \end{matrix} \right.\# (2.3) \\ \mathbb{E}\left[ \sin\left( m\theta\right)\cos\left( n\theta\right) \right]=0 \text{for all }m,n.\# (2.4) \end{aligned}$$

Equivalently, letting $\Sigma_{\phi}:=\text{Cov}\text{(}\Phi_{M}\left( \theta\right)\text{)}$,

$$\begin{aligned} \Sigma_{\phi} = \frac{1}{2}I_{2M}\#\left( 2.5 \right) \end{aligned}$$

When the calendar is not uniform, define the Gram matrix

$$\Sigma_{\phi} := \mathrm{Cov}_{\mu}\left( \Phi_{M}\left( \theta\right) \right) \text{and} \bar{\Phi}:=\mathbb{E}_{\mu}\left[ \Phi_{M}\left( \theta\right) \right]$$

Then the same derivations below hold with $\frac{1}{2}I$ replaced by the empirical (or design-specific) $\Sigma_{\phi}$ and mean $\bar{\Phi}$, see section 2.8.

2.3 **Variance decomposition**

Define the seasonality-adjusted outcome:

$$\tilde{Y}_{ij} := Y_{ij}-\beta_{0}-x_{ij}^{\top}\gamma= \underset{S\left( \theta_{ij} \right)}{\underbrace{\beta_{\phi}^{\top}\Phi_{M}\left( \theta_{ij} \right)}}+\underset{B_{0}}{\underbrace{b_{0i}}}+\underset{B_{\phi}\left( \theta_{ij} \right)}{\underbrace{b_{i,\phi}^{\top}\Phi_{R}\left( \theta_{ij} \right)}}+\varepsilon_{ij}$$

We compute the unconditional variance of $\tilde{Y}_{ij}$ across participants $i$ and the annual cycle $\theta$:

$$\begin{aligned} \mathrm{Var}\left( \tilde{Y} \right) = \mathrm{Var}\left( S\left( \theta\right) \right) + \mathrm{Var}\left( B_{0} \right) + \mathrm{Var}\left( B_{\phi}\left( \theta\right) \right) + \mathrm{Var}\left( \varepsilon\right) + 2\sum_{\mathrm{pairs}} \mathrm{Cov}\left( \cdot,\cdot\right)\#\left( 2.6 \right) \end{aligned}$$

We now show the cross-covariances vanish and each variance term has a closed form.

2.4 **Cross-covariances vanish**

Because $\mathbb{E}\left[ b_{0i} \right]=0$ and $\left( b_{0i},b_{i,\phi} \right)$ are independent of $\theta$:

$$\mathrm{Cov}\left( S\left( \theta\right),B_{0} \right) = \mathbb{E}\left[ S\left( \theta\right)B_{0} \right]\mathbb{-E}\left[ S\left( \theta\right) \right]\mathbb{E}\left[ B_{0} \right] = 0$$

and similarly $\text{Cov}\left( S\left( \theta\right),\varepsilon\right)=0$ and $\text{Cov}\left( B_{0},\varepsilon\right)=0$.

For $\text{Cov}\text{(}S\left( \theta\right),B_{\phi}\left( \theta\right))$:

$$\mathbb{E}\left[ S\left( \theta\right)B_{\phi}\left( \theta\right) \right]=\mathbb{E}_{\theta}\left[ \beta_{\phi}^{\top}\Phi_{M}\left( \theta\right) \mathbb{E}_{b}\left[ b_{i,\phi}^{\top}\Phi_{R}\left( \theta\right) \right] \right]=\mathbb{E}_{\theta}\left[ \beta_{\phi}^{\top}\Phi_{M}\left( \theta\right) \underset{=0}{\overset{\top}{\underbrace{\mathbb{E}_{b}\left[ b_{i,\phi} \right]}}}\Phi_{R}\left( \theta\right) \right]=0$$

Likewise, $\text{Cov}\left( B_{0},B_{\phi}\left( \theta\right) \right)=0$ because $\mathbb{E}\left[ \Phi_{R}\left( \theta\right) \right]=0$ under uniform $\theta$ (or after centering by $\bar{\Phi}$) even if $b_{0i}$ and $b_{i,\phi}$ are correlated.

Hence all cross terms in (2.6) are zero, yielding

$$\begin{aligned} \mathrm{Var}\left( \tilde{Y} \right)=\underset{population seasonality}{\underbrace{\mathrm{Var}\left( S\left( \theta\right) \right)}}+\underset{\mathrm{baseline}\left( between-person \right)}{\underbrace{\mathrm{Var}\left( B_{0} \right)}}+\underset{person-specific seasonality}{\underbrace{\mathrm{Var}\left( B_{\phi}\left( \theta\right) \right)}}+\underset{\mathrm{residual}}{\underbrace{\sigma^{2}}}\#\left( 2.7 \right) \end{aligned}$$

2.5 **Closed-form expressions under uniform calendar**

**Population (fixed) seasonality.**

Since $S\left( \theta\right)=\beta_{\phi}^{\top}\Phi_{M}\left( \theta\right)$ and $\mathbb{E}\left[ \Phi_{M}\left( \theta\right) \right]=0$,

$$\mathrm{Var}\left( S\left( \theta\right) \right)=\beta_{\phi}^{\top}\mathrm{Cov}\left( \Phi_{M}\left( \theta\right) \right) \beta_{\phi}=\beta_{\phi}^{\top}\left( \frac{1}{2}I_{2M} \right)\beta_{\phi}=\frac{1}{2}\sum_{m=1}^{M} \left( \beta_{s,m}^{2}+\beta_{c,m}^{2} \right)$$

where $\beta_{s,m}$ and $\beta_{c,m}$ are the sine and cosine coefficients for harmonic $m$.

**Between-person baseline.**

By definition,

$$\mathrm{Var}\left( B_{0} \right) =\mathrm{Var}\left( b_{0i} \right) = G_{00}$$

the $\left( 0,0 \right)$ entry of the random-effects covariance matrix $G$.

**Person-specific seasonality.**

Write $B_{\phi}\left( \theta\right)=b_{i,\phi}^{\top}\Phi_{R}\left( \theta\right)$. With independence of $b_{i,\phi}$ and $\theta$ and $\mathbb{E}\left[ b_{i,\phi} \right]=0$,

$$\begin{aligned} \mathrm{Var}\left( B_{\phi}\left( \theta\right) \right)&\mathbb{=E}\left[ (b_{i,\phi}^{\top}\Phi_{R}(\theta))^{2} \right] \\ &=\mathbb{E}_{b}\left[ \mathbb{E}_{\theta}[b_{i,\phi}^{\top}\Phi_{R}(\theta)\Phi_{R}(\theta)^{\top}b_{i,\phi} \left| b_{i,\phi} \right] \right] \\ &=\mathbb{E}_{b}\left[ b_{i,\phi}^{\top} \underset{=\frac{1}{2}I_{2R}}{\underbrace{\mathbb{E}_{\theta}\left[ \Phi_{R}(\theta)\Phi_{R}(\theta)^{\top} \right]}} b_{i,\phi} \right] \\ &=\frac{1}{2} \mathbb{E}_{b}\left[ \parallel b_{i,\phi}\parallel_{2}^{2} \right]=\frac{1}{2} \mathrm{tr}\left( \mathrm{Var}\left( b_{i,\phi} \right) \right)=\frac{1}{2}\sum_{k=1}^{2R} G_{\phi,kk} \end{aligned}$$

i.e., half the sum of the marginal variances of the seasonal random slopes. Note that correlations among random slopes do not contribute because cross-harmonic expectations vanish under (2.3)-(2.4).

**Residual.** $\text{Var}\left( \varepsilon\right)=\sigma^{2}$

**Conclusion.**

Under a uniform annual calendar,

$$\begin{aligned} \boxed{\mathrm{Var}\left( Y-\beta_{0}-x^{\top}\gamma\right)=\underset{\mathrm{baseline}}{\underbrace{G_{00}}} + \underset{population seasonality}{\underbrace{\frac{1}{2}\sum_{m=1}^{M} (\beta_{s,m}^{2}+\beta_{c,m}^{2})}} + \underset{person-specific seasonality}{\underbrace{\frac{1}{2}\sum_{k=1}^{2R} G_{\phi,kk}}} + \underset{\mathrm{residual}}{\underbrace{\sigma^{2}}}}\#\left( 2.8 \right) \end{aligned}$$

2.6 **Variance of a sinusoid**

Let $a,b\in\mathbb{R}$ and $\theta\sim\text{Unif}\left[ 0,2\pi\right)$. Then

$$\begin{aligned} \mathrm{Var}\left( a\sin\theta+b\cos\theta\right) = \frac{a^{2}+b^{2}}{2}\#\left( 2.9 \right) \end{aligned}$$

**Proof.** Using the phase-amplitude identity, there exist $R\geq0$ and $\varphi\in\mathbb{R}$ such that

$$a\sin\theta+b\cos\theta= R\sin\left( \theta+\varphi\right), \text{with} R=\sqrt{a^{2}+b^{2}}$$

Since a phase shift does not change the distribution of $\theta$ modulo $2\pi$, $\sin\left( \theta+\varphi\right)$ and $\sin\theta$ equal in distribution, hence

$$\mathrm{Var}\left( a\sin\theta+b\cos\theta\right)=Var\left( R\sin\left( \theta+\varphi\right) \right)=R^{2}\mathrm{Var}\left( \sin\theta\right)=R^{2} \left( \mathbb{E[}\sin^{2}\theta\mathbb{]-E}[sin\theta]^{2} \right)=\frac{R^{2}}{2}=\frac{a^{2}+b^{2}}{2}$$

The same identity holds for $a\sin\left( m\theta\right)+b\cos\left( m\theta\right)$ with any integer $m\geq1$ under a uniform calendar.

2.7 **Participant-specific seasonal amplitude**

For a single harmonic, the seasonal component for participant $i$ can be written as

$$\underset{=:a_{i}}{\underbrace{\left( \beta_{s,1}+b_{s,i,1} \right)}}\sin\theta+ \underset{=:b_{i}}{\underbrace{\left( \beta_{c,1}+b_{c,i,1} \right)}}\cos\theta= R_{i,1}\sin\left( \theta+\varphi_{i,1} \right)$$

with amplitude

$$\begin{aligned} R_{i,1}=\sqrt{(\beta_{s,1}+b_{s,i,1})^{2}+(\beta_{c,1}+b_{c,i,1})^{2}}\#\left( 2.10 \right) \end{aligned}$$

By (2.9), the variance over a uniform year of this first-harmonic component equals $R_{i,1}^{2}/2$. Thus, the median or distribution of $R_{i,1}$ across participants summarizes heterogeneity in seasonal amplitudes.

2.8 **General calendar (non-uniform) and empirical design**

Let $\mu$ be any distribution of $\theta$ (e.g., the empirical distribution of observed measurement dates, possibly with weights). Define

$$\bar{\Phi}:=\mathbb{E}_{\mu}\left[ \Phi_{M}\left( \theta\right) \right], \Sigma_{\phi}:=\mathrm{Cov}_{\mu}\left( \Phi_{M}\left( \theta\right) \right)=\mathbb{E}_{\mu}\left[ (\Phi_{M}(\theta)-\bar{\Phi})(\Phi_{M}(\theta)-\bar{\Phi})^{\top} \right]$$

Centering the basis by $\bar{\Phi}$ (or equivalently subtracting $\beta_{\phi}^{\top}\bar{\Phi}$ from the fixed seasonal mean) yields the following generalization of (8):

$$\begin{aligned} \boxed{\mathrm{Var}\left( Y-\beta_{0}-x^{\top}\gamma\right)=G_{00} + \beta_{\phi}^{\top} \Sigma_{\phi} \beta_{\phi} +tr\left( \Sigma_{\phi,R}G_{\phi} \right) + \sigma^{2}}\#\left( 2.11 \right) \end{aligned}$$

where $\Sigma_{\phi,R}=\text{Co}\text{v}_{\mu}\left( \Phi_{R}\left( \theta\right) \right)$ is the covariance of the random-slope basis (the first $2R$ entries of $\Phi_{M}$) and $G_{\phi}=\text{Var}\left( b_{i,\phi} \right)$ is the $\left( 2R \right)\times\left( 2R \right)$ seasonal block of $G$. This follows from independence of $b_{i,\phi}$ and $\theta$ and the identity

$$\mathrm{Var}\left( b_{i,\phi}^{\top}\Phi_{R}\left( \theta\right) \right)=\mathbb{E}_{\theta}\mathbb{E}_{b}\left[ (b_{i,\phi}^{\top}\Phi_{R}(\theta))^{2} \right]=tr\left( \underset{= \Sigma_{\phi,R}+\bar{\Phi}_{R}\bar{\Phi}_{R}^{\top}}{\underbrace{\mathbb{E[}\Phi_{R}(\theta)\Phi_{R}(\theta)^{\top}]}} \underset{= G_{\phi}}{\underbrace{\mathbb{E}\left[ b_{i,\phi}b_{i,\phi}^{\top} \right]}} \right)-\underset{= 0}{\underbrace{\mathbb{(E[}\Phi_{R}(\theta)]^{\top}\mathbb{E[}b_{i,\phi}])^{2}}}=tr\left( \Sigma_{\phi,R}G_{\phi} \right)$$

since $\mathbb{E}\left[ b_{i,\phi} \right]=0$.

Under a uniform calendar, $\Sigma_{\phi}=\frac{1}{2}I_{2M}$ and $\Sigma_{\phi,R}=\frac{1}{2}I_{2R}$, and (2.11) reduces to (2.8).

Supplementary Note 3. Description of covariates

Age: age in years at accelerometry measurement (field-id: 21022). Sex: self-reported sex was recorded as female or male (field-id: 31). Ethnicity: self-reported ethnicity was categorized as non-White or White (field-id: 21000). Townsend deprivation index: Townsend deprivation index was calculated immediately prior to participant joining UK Biobank, based on the preceding national census output areas. Each participant is assigned a score corresponding to the output area in which their postcode is located (field-id: 22189). Education level: participants reported their education qualifications (field-id: 6138) as college or university degree; A levels, AS levels, or equivalent; O levels, GCSEs, or equivalent; CSEs or equivalent; NVQ, HND, HNC, or equivalent; other professional qualifications; none of the above (equivalent to less than high school diploma); or prefer not to answer (which was excluded as missing values). Smoking status: smoking status (field-id: 20116) was assessed using two questions summarizing current and past smoking behavior (field-id: 1239 and 1249). Individuals who responded to current tobacco smoking with “yes, on most or all days”, or “only occasionally” were classified as current smokers. Individuals who responded to the question regarding past tobacco smoking with “smoked on most or all days” or “smoked occasionally” were classified as previous smokers. Alcohol consumption: alcohol intake frequency (field-id: 1558) was assessed by asking “About how often do you drink alcohol?” Individuals who responded with “never” were classified as none currently. Individuals who responded with “special occasions only”, or “one to three times a month”, or “once or twice a week” were classified as up to twice per week. Individuals who responded with “three or four times a week” or “daily or almost daily” were classified as three or more times per week. Body mass index (BMI): BMI (field-id: 21001) was calculated as weight in kilograms divided by height in meters squared. Healthy diet score: a cumulative diet score was constructed to reflect the diet pattern. A total of 7 food items was used to create the diet score, including the frequency of consumption of fruits, vegetables, fish, processed meat, unprocessed red meat, whole grains, and refined grains^1,2^. The healthy standards for each dietary component were listed as: (1) fruits (field-id: 1309 and 1319): ≥ 3 servings/day; (2) vegetables (field-id: 1289 and 1299): ≥ 3 servings/day; (3) fish (field-id: 1329 and 1339): ≥ 2 servings/week; (4) processed meat (field-id: 1349): ≤ 1 serving/week; (5) unprocessed red meat (field-id: 1369, 1379 and 1389): ≤ 1.5 servings/week; (6) whole grains (field-id: 1438, 1448, 1458 and 1468): ≥ 3 servings/day; (7) refined grains (field-id: 1438, 1448, 1458 and 1468): ≤ 1.5 servings/day. We assigned 1 point for a healthy level and 0 point for an unhealthy level. Finally, a diet score ranging from 0 (least healthy) to 7 (healthiest) was derived by summing the points. Sleep duration: total durations of all sleep episodes within the given sleep period time window (hours per day). Moderate-to-vigorous physical activity: the summed duration of 5-second epochs having a mean acceleration > 100 milli-gravity (hours per day). Sedentary duration: defined as more than 2 consecutive 30-second epochs classified as waking sedentary behaviors (hours per day; sleep was not included in sedentary behavior time)^3^.

Supplementary Note 4. Description of shift workers and medication use

Participants with responses other than “never/rarely” in question job involves shift work (field-id: 826) and job involves night shift work (field-id: 3426) were considered as shift workers. Medication use was assessed using Data-Field 20003, which records nurse-administrated self-reported regular prescription medications. We identified medications using their Anatomical Therapeutic Chemical (ATC) classification system codes^4^ and mapped them to corresponding UK Biobank treatment codes (refer to <https://biobank.ndph.ox.ac.uk/showcase/coding.cgi?id=4>). Benzodiazepines: ATC codes N03AE01, N05BA01, N05BA02, N05BA04, N05BA05, N05BA06, N05BA08, N05BA09, N05BA11, N05BA12, N05BB01, N05BE01, N05CD01, N05CD02, N05CD03, N05CD06, N05CD07, N05CD08, N05CD09, N05CD11, N05CF01, N05CF02, treatment codes 1140872150, 1140863152, 1141157496, 1140863328, 1140863442, 1140863274, 1140863302, 1140863318, 1140863268, 1140855944, 1140863308, 1140883656, 1140879730, 1140863110, 1140863182, 1140863104, 1140863176, 1140863202, 1140863120, 1140863144, 1140865016. Selective serotonin reuptake inhibitors (SSRIs): ATC codes N06AB03, N06AB04, N06AB05, N06AB06, N06AB08, N06AB10, treatment codes 1140879540, 1140921600, 1140867888, 1140867878, 1140879544, 1141180212. Non-selective monoamine reuptake inhibitors: ATC codes N06AA02, N06AA04, N06AA09, N06AA10, N06AA12, N06AA16, N06AA21, treatment codes 1140879630, 1140879620, 1140879616, 1140867818, 1140867640, 1141168396, 1140909806, 1140879552. Mirtazapine: ATC code N06AX11, treatment code 1141152732. Antihistamines for systemic use: ATC codes R06AE03, R06AE06, R06AE07, R06AE09, R06AX02, R06AX12, R06AX13, R06AX15, R06AX17, R06AX18, R06AX22, R06AX25, R06AX26, R06AX27, treatment codes 1140868080, 1140862738, 1140883504, 1141184748, 1140883584, 1140862830, 1141157322, 1140862772, 1141157324, 1140862726, 1140862668, 1140862760, 1141157056, 1141146428, 1141172924. Carboxamide derivatives: ATC codes N03AF01, N03AF02, treatment codes 2038459704, 1140872064, 1141175204. Corticosteroids for systemic use (plain): ATC codes H02AB01, H02AB02, H02AB04, H02AB06, H02AB07, H02AB08, H02AB09, H02AB10, treatment codes 1140874790, 1140874816, 1140874976, 1141157402, 1140874930, 1140868364, 1140868426, 1141157294, 1140874896, 1140884704, 1141173346. Thyroid hormones: ATC code H03AA01, H03AA02, treatment code 1141191044, 1140910520, 1140884512, 1140910518. Insulin: ATC code A10A, treatment code 1140883066. Metformin: ATC codes A10BA02, A10BD02, A10BD03, treatment codes 1140884600, 1141189090. Beta blocking agents: ATC codes C07AA03, C07AA05, C07AA06, C07AA07, C07AB02, C07AB03, C07AB04, C07AB07, C07AB08, C07AB12, C07BB02, C07BB03, C07BB07, C07CB03, treatment codes 1140860292, 1140879842, 1140879866, 1140879854, 1140879818, 1140866738, 1140866724, 1140879760, 1140879762, 1141164276, 1140860404, 1140864950, 1140866738. Statins: ATC codes C10AA01, C10AA04, C10AA05, C10BA02, C10AA03, C10AA07, treatment code 1140861958, 1140888594, 1141146234, 1140888648, 1141192410. Nonsteroidal anti-inflammatory drugs (NSAIDs): ATC code M01AB01, M01AB02, M01AB05, M01AB16, M01AB55, M01AC01, M01AC06, M01AE01, M01AE02, M01AE03, M01AE11, M01AE14, M01AH05, treatment code 1141181656, 1140909936, 1140871604, 1140884488, 1140925806, 1140884488, 1140871666, 1140926732, 1140871310, 1141157412, 1140871462, 1140871506, 1140875642, 1140871614, 1141180140.

Supplementary Figure 1 Flow chart for accelerometer data quality control

Supplementary Figure 2 Distribution of alignment features

**a-d**, histogram of peak lag hour $\tau_{\mathrm{peak}}$ of 24-h cross-correlation $r_{xy}\left[ \tau\right]$, M_24_, M_12_ and D_24_, respectively

Supplementary Figure 3 Comparisons between features derived from bootstrap and the original method

Supplementary Figure 4 Comparisons between recomputing features and the original features

Supplementary Figure 5 Comparisons between features removing seasonality and the original features

Supplementary Figure 6 Cross-spectral decomposition of circular cross-correlation across diurnal harmonics

Blue bars (left *y*-axis) show the cohort median energy share for each harmonic *k* (excluding *k* = 0), and the orange line with dots (right *y*-axis) shows the proportion of participants with significant coupling at that harmonic (permutation test with BH-FDR *q* < 0.05).

Supplementary Figure 7 Potential nonlinear associations between alignment features and disease phenotypes

Supplementary Figure 8 Comparisons between sensitivity analyses and the original PheWAS analyses

Supplementary Figure 9 Technical validation of alignment features in the SHARE cohort

**a-d**, histogram of peak lag hour $\tau_{\mathrm{peak}}$ of 24-h cross-correlation $r_{xy}\left[ \tau\right]$, M_24_, M_12_ and D_24_, respectively

Supplementary Figure 10 Distribution of participant-level completeness indicators in the UK Biobank

Supplementary Figure 11 Distribution of participant-level completeness indicators in the SHARE

Supplementary Figure 12 Comparisons between high-completeness subset and the original PheWAS associations

Supplementary Figure 13 Comparisons between completeness-indicators adjustment and the original PheWAS associations

Supplementary Figure 14 Comparisons between acceleration-spike winsorization and the original PheWAS associations

Supplementary Figure 15 Comparisons between baseline sleep-related traits adjustment and the original PheWAS associations

Supplementary Figure 16 Distribution of higher harmonic components

References

1.	Mozaffarian, D. Dietary and Policy Priorities for Cardiovascular Disease, Diabetes, and Obesity: A Comprehensive Review. *Circulation* **133**, 187–225 (2016).

2.	Morris, M. C. *et al.* MIND diet associated with reduced incidence of Alzheimer’s disease. *Alzheimers Dement.* **11**, 1007–1014 (2015).

3.	Raichlen, D. A. *et al.* Sedentary Behavior and Incident Dementia Among Older Adults. *JAMA* **330**, 934 (2023).

4.	Liu, J. *et al.* Integration of epidemiologic, pharmacologic, genetic and gut microbiome data in a drug–metabolite atlas. *Nat. Med.* **26**, 110–117 (2020).
